# Effect of total population, population density and weighted population density on the spread of Covid-19 in Malaysia

**DOI:** 10.1371/journal.pone.0284157

**Published:** 2023-04-27

**Authors:** Hui Shan Wong, Md Zobaer Hasan, Omar Sharif, Azizur Rahman

**Affiliations:** 1 School of Science, Monash University Malaysia, Jalan Lagoon Selatan, Bandar Sunway, Selangor D. E., Malaysia; 2 General Educational Development, Daffodil International University, Daffodil Smart City, Ashulia, Dhaka, Bangladesh; 3 Department of Mathematics and Statistical Science, University of Texas Rio Grande Valley, Edinburg, Texas, United States of America; 4 School of Computing, Mathematics and Engineering, Charles Sturt University, Wagga Wagga, Australia; Nanyang Technological University, SINGAPORE

## Abstract

Since November 2019, most countries across the globe have suffered from the disastrous consequences of the Covid-19 pandemic which redefined every aspect of human life. Given the inevitable spread and transmission of the virus, it is critical to acknowledge the factors that catalyse transmission of the disease. This research investigates the relation of the external demographic parameters such as total population, population density and weighted population density on the spread of Covid-19 in Malaysia. Pearson correlation and simple linear regression were utilized to identify the relation between the population-related variables and the spread of Covid-19 in Malaysia using data from 15^th^ March 2020 to 31^st^ March 2021. As a result, a strong positive significant correlation between the total population and Covid-19 cases was found. However, a weak positive relationship was found between the density variable (population density and weighted population density) and the spread of Covid-19. Our findings suggest that the transmission of Covid-19 during lockdown (Movement Control Order, MCO) in Malaysia was more readily explained by the demographic variable population size, than population density or weighted population density. Thus, this study could be helpful in intervention planning and managing future virus outbreaks in Malaysia.

## Introduction

The coronavirus disease 2019 (Covid-19) is a contagious disease caused by Severe Acute Respiratory Syndrome Coronavirus 2 emerged in Wuhan, China at the end of December 2019 [1]. It was found to be first transmitted from an animal host to humans in Wuhan, China. As of 20^th^ March 2020, the World Health Organization (WHO) declared Covid-19 a pandemic- a public health emergency of international concern and a green light publication to the scientific, public health, and global [2]. WHO also issued comprehensive guidelines that advised all countries on testing, handling and managing the potential case of Covid-19. As of the current time of this article, Covid-19 caused 2,409,011 deaths and 109,068,745 confirmed cases with the virus spreading to 219 countries around the globe and thus knowledge of the possible Covid-19 catalyst must be known to battle this pandemic [2].

Studies have been conducted in Malaysia to investigate the possible Covid-19 transmission catalyst. For instance, the spread of Covid-19 was positively correlated with Particulate Matter (PM) 10, PM 2.5, sulphur oxide, nitrogen dioxide, carbon dioxide, and relative humidity [3]. The researchers [4] described the clinical characteristics of Covid-19 cases in Malaysia and identified risk factors associated with severe Covid-19 cases. A study [5] found that a massive transmission of Covid-19 infections was caused by a single mass gathering that took place in Sri Petaling from 27 February 2020 to 1 March 2020, with more than 35% of the Covid-19 cases in Malaysia were directly associated, suggesting that mass gathering is one of the catalysts for the spread of Covid-19 infection. States with higher population density had a higher initial transmission of Covid-19. Higher population density areas experienced a quicker decline in the transmission rate after implementing the Movement Control Order in Malaysia [6]. However, in their study they only focused on one demographic variable, population density.

Studies outside Malaysia examine the link between demographic variables on the propagation of Covid-19 transmission, which shows that the relationship is positive in most of the countries. For example, researchers found a significant positive significant relationship between population density and spread of Covid-19 cases in the United States [7], India [8], Algeria [9] and Turkey [10]. Notably, the researchers [11] found that higher income areas had more extensive cases, suggesting that dining out, entertaining and socialization also generate higher infection risk.

Conversely, researchers had also found an insignificant relationship between population density and Covid-19 transmission. A study [12] found a slight connection between Covid-19 transmission and population density in China during the lockdown period. However, they suggested that the lockdown policies of China can decrease human-to-human transmission, which could be an effective measurement for other countries to adhere to in their situation. A paper [13] found an insignificant positive association of population size, temperature and median age on the Covid-19 outbreak. However, the interaction between the three variables showed a significant impact on the spread of Covid-19. Similarly, another study [14] found that only the population density factor was not statistically significant on a country level. The heterogeneity can explain it in data of individual countries as different health measures were being implemented (partial lockdown, full lockdown, no quarantine). These findings suggest that the association between demographic variables and Covid-19 cases in each country should be investigated on a city and/or state-level.

Therefore, there is an urge to acknowledge the factors catalyzing Covid-19 transmission which motivated several researchers to focus on the relationship between demographic factors (population and density) and Covid-19. Recently, a few studies have investigated the effect of such demographic variables on the spread and severity of Covid-19 in Malaysia. For example, a study [15] was conducted to determine the correlation between population density and Covid-19 incidence across the 144 districts within five regions in Malaysia and concluded that population density was an important factor in spreading Covid-19 in Malaysia. However, this study only focused on Malaysia’s third wave of the pandemic for a very short time frame (22 January 2021–4 February 2021). Another paper [16] examined the impacts of population density on the spread and severity of Covid-19 in Malaysia and noticed that population density had a moderately strong relationship with cumulative Covid-19 cases and a weak relationship with Covid-19 infection rates. A research [17] considered three major factors (social, economy and environment) and investigated their effects on the Covid-19 pandemic situation in Malaysia. One of the variables under the social factor was population density, and they identified a positive relationship between population density and Covid-19 infection rate in Malaysia. There was a study [18] which used two population-related demographic variables and studied their impacts on the incidence and distribution of Covid-19 cases in Malaysia at the district level. Based on the study findings, the researchers concluded that more populous and densely populated districts had a higher risk of transmission of Covid-19, especially with the delta variant.

Based on our literature review search, there is no such study in Malaysia which use one more important population-related demographic variable, weighted population density and examine its effect on Covid-19 spread in Malaysia. Hence, this research aims to extend the knowledge of the possible demographic parameters that can catalyse the spread of the virus in Malaysia. Thus, this research will be the first study that empirically evaluates the effect of the weighted population density along with the total population, population density on Covid-19 transmission on a state-level in Malaysia. Therefore, these demographic factors cannot be discounted in the research of transmission of the disease for the intervention planning and managing future outbreaks.

## Material and methods

### Data sources

Malaysia is divided into 13 states; Sabah, Melaka, Johor, Sarawak, Perak, Pulau Pinang, Pahang, Kedah, Terengganu, Selangor, Negeri Sembilan, Perlis and 3 federal territories; Wilayah Persekutuan (WP) Putrajaya, WP Kuala Lumpur, WP Labuan. In this study, the secondary data were used as input for analyses and we used three independent variables (total population, population density and weighted population density) and one dependent variable (Covid-19 cases).

First, day-wise data for the cumulative number of Covid-19 cases of 13 states and three federal territories from 15 March 2020 to 31 March 2021 were acquired from the website of the Ministry of Health (MOH) [19].

Second, the total population and total area data of all 13 states and three federal territories were obtained from the official report from the Department of Statistics Malaysia [20]. These aggregated data were then used to calculate population density for each state and federal territory, the number of individuals inhabiting an area of 1 km^2^ [9]. Hence, the population density was calculated by using the following equation:

$$D_{p}=\frac{N}{A}$$ (1)

where

*D*_*P*_ = Population density of the state/territory

*N* = Total number of individuals (population size) under the state/territory

*A* = Total area of the state/territory

Thirdly, to calculate the weighted population density of different states and territories, the district wise population and their respective area of all the 13 states and one federal territory (except for WP Labuan and WP Putrajaya) were obtained from the demographic report of the Department of Statistics Malaysia [20]. The reason for excluding the two territories in calculating weighted population density was that WP Labuan and WP Putrajaya are considered a single district in Malaysia. The weighted population density of each state and federal territory, which is regarded as a more accurate estimate of the standard population density was determined by using the following formula [21]:

$$WD_{p}=\overset{M}{\underset{i=1}{\sum }}(\frac{n_{i}}{n})(\frac{n_{i}}{A_{i}})$$ (2)

where

*WD*_*p*_ = Weighted population density of the state/territory

*n*_*i*_ is the district-wise population under each state/territory

*n* is the total population of the state/territory

*A*_*i*_ is the district-wise area under each state/territory

M is the total number of districts in the state/territory

### Statistical analysis

To see the influence of total population, population density and weighted population density on the transmission of Covid-19 in Malaysia, two statistical analyses, Pearson correlation and simple linear regression were conducted using IBM SPSS Statistics 26. In this study, the statistical significance p-value less than or equal to 0.05 was used.

### Pearson correlation

Pearson correlation coefficient was utilized in this study as it is the most appropriate statistical method to examine the relation and strength nature of the two quantitative variables [22]. The Pearson correlation equation is as follows [23].


$$r=\frac{\frac{1}{N}\sum xy-\bar{x}\bar{y}}{\sqrt{(\frac{1}{N}\sum x^{2}-{\bar{x}}^{2})\sqrt{\left(\frac{1}{N}\sum y^{2}-{\bar{y}}^{2}\right)}}}$$ (3)

Such as:

*r* = Pearson’s product moment correlation coefficient

*N* = number of pairs of values or scores

∑*xy* = Sum of products of *x* and *y*

$\bar{x}$ = mean of *x* values

$\bar{y}$ = mean of *y* values

∑*x*^2^ = sum of squares of *x* values

∑*y*^2^ = sum of squares of *y* values

To examine the strength of the correlation, this study used the benchmark of [24].

### Selection of regression model

The definition of the regression model describes the association between quantitative unknowns by fitting a line to the set of data. Moreover, a simple linear regression model is applied to find “How strong the relationship is between two unknowns”. However, before selecting the model, this study considered three issues in variables: perfect reference, bivariate case, and global versus localized measure. This research mainly aimed to show the relationship of bivariate variables where the data had no missing value and were not containing any error. Therefore, the simple linear regression model was applied in this study because it is a suitable model for comparing two unknowns that do not contain any missing or error value [25]. There are some other regression models that have also been applied for bivariate case comparisons. For example, unreplicated linear functional relationship [26], multiple linear regression [27], logistic regression [28] and canonical correlation [29]. Hence, the following table suggests why the simple linear regression model is suitable for this study. Table 1 shows the comparison of some well-known regression models.

**Table 1:** Comparing well-known regression models.

Variables	Unreplicated linear functional relationship (ULFR)	Multiple correlation	COD for logistic regression	Canonical correlation	Linear regression
**Y subject to error**	Yes	Yes	Yes	No	No
**X subject to error**	Yes	No	NO	NO	No
**Multivariate for X**	Yes	Yes	Yes	Yes	No
**Multivariate for Y**	Yes	No	No	Yes	No

### Simple linear regression

A simple linear regression model was constructed to determine the effect of total population, population density and weighted population density on the spread of Covid-19 infection in the 13 states and three federal territories. Hence, the sample size of 16 was used for the regression calculation. The linear model is as given below:

y=a+bx (4)

Such as:

*y* = dependent variable that represents the cumulative number of Covid-19 daily cases

*x* = independent variable that represents the three studied variables (total population/population density/weighted population density)

*a* = constant

*b* = slope

Moreover, the coefficient of determination (*R*^2^) was used in this study to assess the degree of fit of the model. The value of *R*^2^ indicates the variation in the response is explained by the model. More specifically, the interpretation of *R*^2^ is the fraction of variance described by the regression model. The equation of *R*^2^ is as follows [30]:

$$R^{2}=1-\frac{SSR}{SST}=1-\frac{\sum {\left(y_{i}-{\hat{y}}_{l}\right)}^{2}}{{\left(y_{i}-{\bar{y}}_{l}\right)}^{2}}$$ (5)

Whereby

*SSR* = Regression Sum of Squares

*SST* = Total Sum of Squares

*y*_*i*_ = Actual Value

$\hat{y_{i}}$ = Predicted Value of *y*

$\bar{y_{i}}$ = Mean Value of *y*

There may have some uncertainties in the regression models. Based on [31], the uncertainties occur due to noise and imperfect fit to the historical data. However, adjusting uncertainty is a crucial procedure [32] and most journal papers used the regression model but did not apply uncertainty rules.

## Results and discussion

The research aimed to investigate the effect of the total population, population density and weighted population density on the spread of Covid-19 cases in Malaysia on a state and federal territory level by using two statistical analyses; Pearson correlation and simple linear regression.

### Preliminary results of the study

Table 2 contains the summary statistics of the variables: cumulative Covid-19 cases, total population, population density and weighted population density.

**Table 2:** Summary statistics of the studied variables.

Variables	Sample size	Mean	Standard deviation	Minimum	Maximum
**Cumulative Covid-19 cases**	16	21593.75	29809.75	330	116078
**Total population**	16	2041093.75	1657986.10	99600	6538100
**Population density**	16	941.25	1814.90	22.63	7299.18
**Weighted population density**	14	1313.07	2071.48	89.84	8054.06

The names of 13 states and three federal territories of Malaysia with cumulative Covid-19 cases in percentages and actual numbers are provided in the appendix. As seen in the appendix, Selangor has the highest number of Covid-19 cases, contributing to 33.6% of the total cumulative Covid-19 cases in Malaysia and Sabah comes second with contributing 15.85% of the overall Covid-19 cases in Malaysia. On the contrary, Perlis has the lowest cumulative Covid-19 cases contributing 0.1% of cases in Malaysia with only 330 cases.

**Fig 1:**
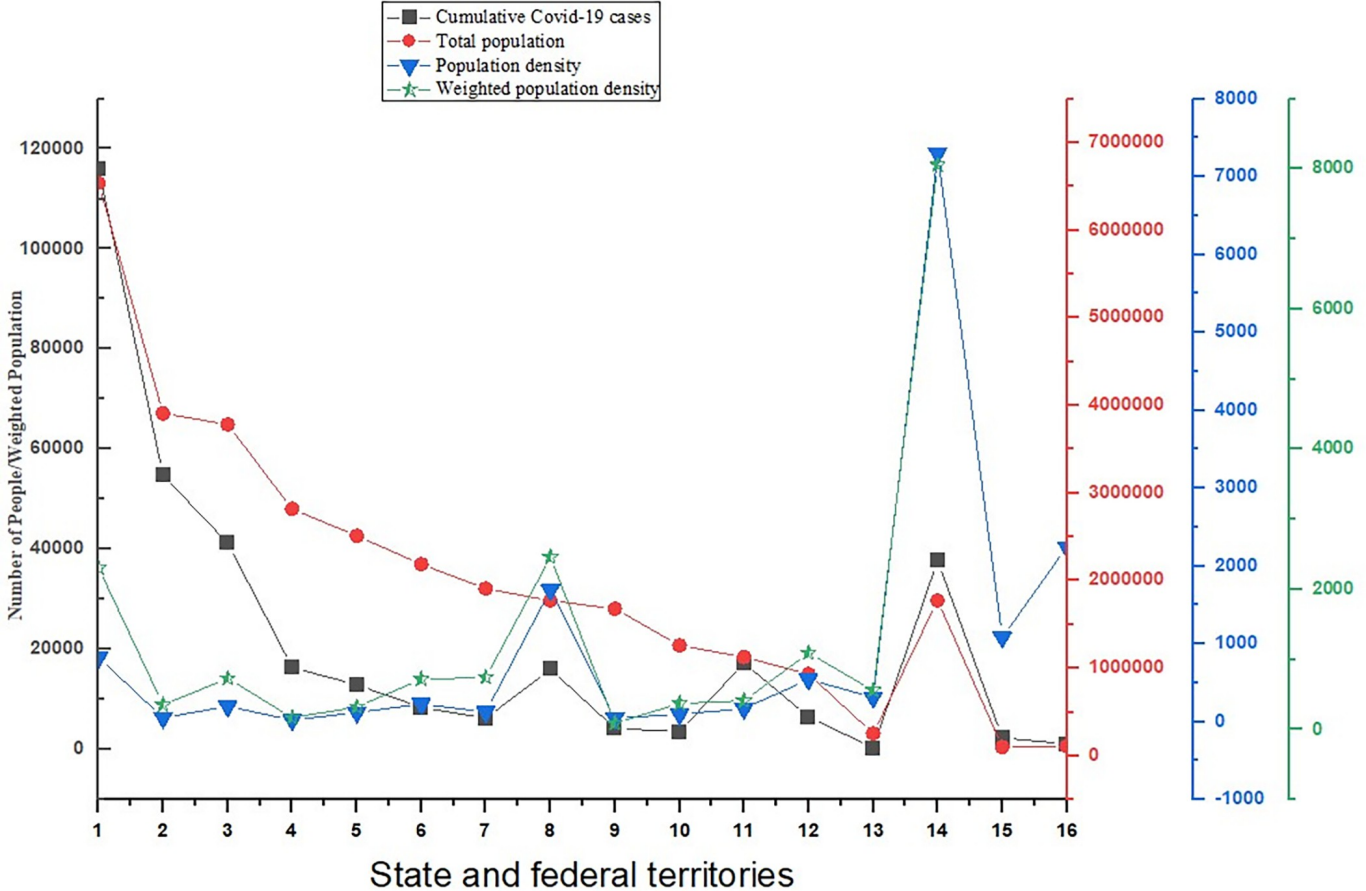
Distribution of Covid-19 cases, total population, population density, and weighted population density [The weighted population densities for the last two territories are considered the same as their respective population densities].

In Fig 1, the distributions of Covid-19 cases with the total population, population density, and weighted population density are provided according to the 13 states and three federal territories. It is clearly visible that the total population has a strong positive correlation with Covid-19 cases as black and red lines approximately coincided. However, a weak positive correlation is found in the density variables with Covid-19 cases because the blue and green lines do not exactly coincide with the black line. It can also be seen that Selangor (1) has the highest total population but the second highest in total cumulative Covid-19 cases. Sabah (2) with the lowest population density, but the highest number of cumulative Covid-19 cases. WP Kuala Lumpur (14) with the highest population density but is third in place for cumulative Covid-19 cases. Pulau Pinang (8) with the highest weighted population density but with relatively low cumulative Covid-19 cases.

### Correlation and regression analysis of total population and Covid-19 cases

As per Table 3, positive and very strong correlation (*r* = 0.899) was found between the two variables, total population and Covid-19 cases. And, the relationship was significant at 5% significance level (p-value < 0.05). These findings were consistent with [18], which investigated the correlation between absolute population and Covid-19 cases in Malaysia at the district level and found a significant strong positive correlation (*r* = 0.87). A study [7] form United States examined the direct and indirect factors on Covid-19 cases and mortality rates in 913 Metropolitan using structural equation modelling. They found a positive correlation (*r* = 0.285) between the total population in the metropolitan area and Covid-19 transmission rate. They also noticed that total population is one of the most important predictors of the Covid-19 infection among all the other studied factors. A similar study of [14] found that the relation between total population and Covid-19 cases are positive and significant for the countries; USA (*r* = 0.4342, p-value = 0.0015), Spain (*r* = 0.7271, p-value = 0.0004), Germany (*r* = 0.9160, p-value <0.001) and United Kingdom (*r* = 0.8016, p-value = 0.0017). In terms of our study, Selangor has the highest total population in Malaysia and during the write-up, it has the highest cumulative Covid-19 cases in Malaysia. Contrastingly, the study [13] investigated the relationship between population size and Covid-19 transmission of the top 58 countries in terms of confirmed cases and found an insignificant correlation at the 5% level of significance (p-value = 0.250). However, they found that the interactions of population size and other variables such as temperature and median age significantly affect the spread of Covid-19 (p-value = 0.001). Thus, it can be suggested that a single individual factor of population size does not confer a significant impact on the spread of Covid-19 but interaction among other factors could express a more significant impact, which can be a direction for future studies and research.

**Table 3:** Pearson correlation and simple linear regression analysis for total population and cumulative Covid-19 cases.

Regression with Intercept	Findings	p-value
**Pearson correlation (*r*)**	0.899	2.00E-06
**Intercept (*a*)**	-11380.533	0.057
**Slope (*b*)**	0.016	2.2618E-06
**Coefficient of determination (*R*** ^**2**^ **)**	0.807	
**Regression without Intercept**	**Findings**	**p-value**
**Slope (*b*)**	0.013	2.53E-07
**Coefficient of determination (*R*** ^**2**^ **)**	0.838	

We fitted a simple linear regression model with an intercept, and the value of coefficient of determination (*R*^2^) was 0.807 (Table 3). This value suggested that the total population factor explained the spread of Covid-19 transmission at a rate of 80.7%. Therefore, our findings support that the total population is closely related to the transmission of Covid-19 in the data context of Malaysia. Hence, government bodies and policy makers should focus on cities and/or states with a large total population. It is an extremely important factor in transmitting the disease and inducing an outbreak. Therefore, more restrictions and enforcement of the state of emergency acts should be strictly implemented to control the spread of the disease, especially in states with a high total population which can help mitigate the Covid-19 epidemic.

The regression (with and without intercept) model between total population and Covid-19 cases are given below in Eqs 6 and 7, respectively:

Covid−19cases=−11380.533+0.016(totalpopulation) (6)

Covid−19cases=0.013(totalpopulation) (7)

From Eq 6, it is noticed that the intercept value is negative which is not rational in this case. And, this value was statistically insignificant (Table 3) as well. Hence, we focused on the regression model without intercept and found that this later model is better than the regression model with intercept in terms of *R*^2^ value and p-value for the slope term. Therefore, Eq 7 could be used to predict the future value of the number of Covid-19 infected people. The p-value of the slope coefficient suggests that the total population had a significant effect on the Covid-19 spared in Malaysia. Hence, the value of slope coefficient was 0.013; which explains that for every one-unit increase in total population, there is a corresponding 0.013-unit increase in Covid-19 cases. The larger the population size, the higher the spread of Covid-19. Therefore, the government and health departments can use this model to take precautionary measures before spreading the disease.

### Correlation and regression analysis of population density and Covid-19 cases

The present study showed a positive but very weak correlation (*r* = 0.125) between the two variables, population density and Covid-19 cases and the relationship is insignificant at the 5% level of significance (p-value = 0.645) (Table 4). These findings contradicted [15] and [18], where they reported a significantly strong relationship (*r* = 0.784 and *r* = 0.778) between population density and Covid-19 cases in Malaysia. Two other studies [16,17] in Malaysia found a significantly moderate strong relation (*r* = 0.644 and *r* = 0.390) between these two variables. A research [9] in outside Malaysia studied the effect of population density on the transmission of Covid-19 in 48 Algerian cities and found a moderate and significant correlation (*r =* 0.711) at the 5% level of significance. Another study [8] reported that infection rates are higher in metropolitan cities with large population density in India as they found a fair and highly significant correlation (*r* = 0.49, p-value ~ 10^−37^) at the 5% level of significance. However, some uncertainty was noted in their study and they suggested using weighted population density in future studies.

**Table 4:** Pearson correlation and simple linear regression analysis for population density and cumulative Covid-19 cases.

	Findings	p-value
**Pearson correlation (*r*)**	0.125	0.645
**Intercept (*a*)**	19662.57	0.040
**Slope (*b*)**	2.052	0.645
**Coefficient of *determination* (*R*** ^**2**^ **)**	0.016	

Further, the simple linear regression analysis was conducted for these two variables and the coefficient of determination (*R*^2^) was calculated as 0.016. It suggested that the population density factor only explains the spread of Covid-19 in Malaysia at a rate of 1.6%, and the rest (98.4%) is explained by other factors (Table 4). For instance, Covid-19 cases can be explained by other factors such as air pollution, relative humidity, pre-existing health conditions and diseases hypertension, diabetes, asthma, cardiovascular disease, chronic kidney disease and mass gathering [3–5].

The regression model equation between population density and Covid-19 cases is as follows:

Covid−19cases=19662.57+2.052(populationdensity) (8)

Moreover, the p-value of the slope coefficient was 0.645 which means that the population density had an insignificant effect on the Covid-19 spread in Malaysia.

For instance, Sabah has the lowest population density in Malaysia but the second highest number of cumulative Covid-19 cases as seen in the appendix table. One of the probable reasons is the Sabah state election that took place on 26 September 2020 amid the pandemic. Many citizens and political figures flew over to Sabah to carry out their duty to vote and support their respective parties [33]. During the campaign and voting period, the government permitted inter-state travel and cancellation of mandatory quarantine measures of 14 days for those returning to and from Sabah. Cumulatively, all these factors created an ideal environment for rapid transmission and therefore 31 new clusters were reported which was directly linked to the Sabah state elections [34]. Similarly, in Thailand, the number of tourists was significantly associated with the number of infected cases [35].

Hence, it is evident that implementations such as travel restrictions, quarantine, prohibition of mass gathering, and social distancing are effective measures to reduce population mobility and stop the spread of the Covid-19 virus.

### Correlation and regression analysis of weighted population density and Covid-19 cases

Finally, another type of population density, the weighted population density, was examined as a factor to have a clearer understanding of the impact of population density on Covid-19 cases which was suggested by other researchers [8]. Hence, the weighted population density considers the district’s total population and its total area under each state/territory, which is deemed to be a much more accurate measure than population density. However, a weak positive correlation (*r* = 0.296) was found between the variables weighted population density and Covid-19 cases in the context of Malaysia, and the relationship between these two variables was statistically insignificant (p-value = 0.305) which contradicted with the claim of [21,36,37]. A study [34] noticed a positive and significant relationship between weighted population density and spread of Covid-19 in Turkey (*r* = 0.67, p-value = 0.001). However, he also mentioned that the Turkish government did not limit the citizens to leave their houses; hence, cities with a large population had a higher number of cases. Similarly, researchers in the United States also reported a weak but statistically significant relationship between weighted population density and Covid-19 cases in urban counties (*r* = 0.237, p-value < 0.01) [37]. Nevertheless, it was found that this type of relationship is associated with only the initial stage of Covid-19 arrival. In other words, cities with high population density get hit first but not necessarily harder. The mechanisms that may explain such findings are that cities are intensely interconnected with other locations, explaining the early onset of Covid-19 [37].

Our research analyzed population density and weighted population density on Covid-19 transmission in Malaysia showed a weak correlation. The lack of statistical significance is probably due to the implementation of movement restrictions enforced in Malaysia. The first stage of movement restriction order, Movement Control Order (MCO) was implemented from 18^th^ March to 3^rd^ May 2020, consecutively Conditional Movement Control Order (CMCO) (4^th^ May to 9^th^ June 2020) and Recovery Movement Control Order (RMCO) [33]. During these phases of movement control orders, mass gathering, and movement were prohibited [33], business premises were closed except for daily essential sector, all borders (within states and abroad) and schools was closed, social distancing and only the head of the household was allowed to travel within a certain radius was implemented [38]. In short, everyone was urged to stay at home during the phases of restriction order which limited population mobility. Thus, population density is less critical factor for the spread of Covid-19 in Malaysia during MCO. Lockdown interventions, including social distancing and travel restrictions, reduce population mobility which can mitigate the current Covid-19 pandemic [39]. A study [40] has revealed that Malaysia had successfully reduced the Covid-19 case rates during MCO 1.0. Next, [41] found a significant reduction in Covid-19 cases following the implementation of MCO in the second most populous district in Selangor, called Hulu Langat, which was classified as a Covid-19 red zone. Furthermore, [42] discovered the positive impact of “Enhanced MCO” (EMCO) initially implemented in Bandar Baru Ibrahim Majid, Johor, which was executed along with the cooperation of the police and armed forces, and prohibition of residents from leaving their homes. Although the success of MCO could have indicated the end of the peak, it does not imply Covid-19 eradication and raised concerns on a potential resurgence in transmission after MCO was discontinued. Accordingly, Malaysia was hit by a third outbreak wave in early October 2020 [43]. Moreover, lockdowns implemented across countries have demonstrated variable effectiveness in reducing the transmission of Covid-19 [44]. These contradictory findings could be attributed to differences in timing, duration, and strictness of imposed lockdown measures [44].

Regression analysis was also conducted for weighted population density and Covid-19 cases. The regression model of these two variables is given below:

Covid−19cases=18637.39+4.412(weightedpopulationdensity) (9)

Here, the p-value of the slope coefficient was 0.305. Therefore, like population density, weighted population density also had no significant effect on the Covid-19 spread in Malaysia.

The coefficient of determination (*R*^2^) value obtained was 0.087 (Table 5), higher than the *R*^2^ value of population density and Covid-19 cases. So, this value indicates that the weighted population density factor explains the spread of Covid-19 transmission at a rate of 8.7%. And, the rest (approximately 91%) is explained by other factors. One prominent aspect that has been found in many studies to influence the transmission rate of this disease is from the geographical point of view, or in other words, the geographical factors of an area [45]. Some geographical factors significantly influence the daily Covid-19 new cases in some of Malaysia’s states/federal territories. For instance, the temperature was positively associated with the Covid-19 spreading rate [46] as well as the absolute humidity showed a significant positive association with the spreading rate [46]. Better air quality affected by wind speed in the area also reduced spreading rates, which is particularly evident in coastal areas of Malaysia [47]. However, geographical factors alone do not fully influence how Covid-19 spreads in Malaysia. Various other aspects do play essential roles in influencing Covid-19 transmission. One of the first Malaysian nationwide observational studies showed that the factors associated with severe Covid-19 were ages above 51 years with underlying comorbidities [48]. On the other hand, [49] investigated Covid-19 patients in Sarawak, Malaysia, with underlying conditions of rheumatic diseases and concluded that a history of the rheumatic disease was not a factor that influenced mortality rate or susceptibility to Covid-19 infection. Few studies in Malaysia also looked into the characteristics of the virus itself. [50] conducted a study focusing on a molecular approach by discussing the genomic analysis of isolated strains found in Malaysia. Their study concluded that more than one type of Covid-19 strain is currently present in Malaysia.

**Table 5:** Pearson correlation and simple linear regression analysis for weighted population density and cumulative Covid-19 cases.

	Findings	p-value
**Pearson correlation (*r*)**	0.296	0.305
**Intercept (*a*)**	18637.39	0.082
**Slope (*b*)**	4.412	0.305
**Coefficient of determination (*R*** ^**2**^ **)**	0.087	

Our findings for population density and weighted population density can be supported by the study conducted in China, where they reported that under strict lockdown policies, population density factor might be limited in spreading Covid-19 in China [12]. As they found an insignificant relationship between Covid-19 transmission and population density in China during the lockdown period. This result suggests that the implementation of strict lockdown is highly effective even in areas with high population density. In addition, a study conducted in Hubei, China, found a significant relationship between the population mobility and Covid-19 cases, (p-value < 0.05) [51]. The authors showed that the daily confirmed cases and daily increment incidence of Covid-19 have declined after the implementation of city lockdown. It suggests that strategies such as restricting population mobility have effectively curbed the spread of Covid-19 transmission in Hubei, China, which can be a valuable strategy for controlling the spread of the virus in Malaysia.

Based on our literature review search, there was no study related to the impact of the demographic variables on the spread of Covid-19 using any non-linear models. However, some studies used non-linear models to deal with the relationship between Covid-19 and some non-demographic variables. For example, [52] identified a significant non-linear link between temperature difference and Covid-19 in the case of the United States. Another study [53] focused on 65 countries of the world and examined the nonlinear relationship between Covid-19 cases and carbon damages, managing financial development, renewable energy consumption, and innovative capability. A complete set of non-linear modelling, including the quantile-on-quantile (QQ) regression and quantile Granger causality in mean was applied to examine the asymmetric inter-linkages between transportation mobility and Covid-19 in ten selected countries (i.e., USA, Brazil, Mexico, UK, Spain, Italy, France, Germany, Canada, and Belgium) [54]. A study [55] used more than a hundred countries’ data and performed a comparative graphical analysis with non-linear correlation estimation to analyze the outcome of Covid-19 in response to different control measures, healthcare facilities, life expectancy, and prevalent diseases. They used these non-linear models because many of these studies found that the linear (direct) relationship is not apparent.

Overall, this study empirically proved that the main demographic parameter influencing the spread of Covid-19 in Malaysia is total population, as population density and weighted population density had little to no effect on the transmission of Covid-19. Our findings also support and justify the policy of implementing specific lockdown interventions in states and/or cities with large population sizes. The recommendations provided by this study will be valuable in intervention planning and imposing preventive measures for future virus outbreaks in Malaysia and help better prepare for such situations.

## Conclusions

In this research, we investigated the impact of demographic variables: total population, population density, and weighted population density on the spread of Covid-19 in Malaysia as well as tried to identify which demographic variables are most important in the spread of the Covid-19 pandemic in Malaysia. The relationship between the spread of Covid-19 and different predictors has been widely debated in scientific journals, magazines, and reports around the world [56]. In our research, the obtained results indicate that the total population variable is the most important variable among the studied three demographic variables and has a positive significant relationship with Covid-19 transmission in Malaysia’s different states and federal territories. The larger the population size, the higher the spread of Covid-19. Therefore, our findings suggest that states and/or cities with a higher population size should have emphasized stricter and more precise policies to curb the spread of Covid-19. Conversely, both types of density variables (population density and weighted population density) have an insignificant relationship with Covid-19 transmission in Malaysia. Even though the pattern of our findings is not consensual worldwide (i.e., there are studies that report density or weighted density as an important predictor due to the multifactorial nature of Covid-19 and the interaction between the different factors [9,34]), it aligns with some of the published research [7,56,57]. Furthermore, as the density variable holds complementary information to the total population; it could be considered in the different epidemiological models [56].

Nonetheless, these results must be interpreted with caution and should note a few limitations. First, the cumulative data of Covid-19 cases are limited to 15^th^ March 2020 to 31^st^ March 2021, which is only a year’s worth of data. Therefore, it is recommended that further studies with long-duration data be undertaken to obtain a clear relationship between the demographic variables and the spread of Covid-19 in Malaysia. And, the simple linear regression is carried out using a small sample, therefore the findings need to be interpreted carefully. In addition, more elaborate statistical tools can be used, which may lead to different conclusions than simple linear regression. Another limitation would be the lack of data for two federal territories, Labuan and Putrajaya, to calculate weighted population density. Thus, the weighted population density was unable to be calculated for the whole of Malaysia. Moreover, the daily confirmed Covid-19 cases reflect the cases detected rather than case infected; hence there may be a measuring error as not every Covid-19 case was confirmed and reported immediately.

## Supporting information

S1 File (XLSX)

S2 File (XLSX)

S1 Appendix Data of studied research. (DOCX)
