# Supplementary material for: Effect of total population, population density and weighted population density on the spread of Covid-19 in Malaysia
Source: PLoS One. 2023 Apr 27;18(4):e0284157. doi: 10.1371/journal.pone.0284157 (PMC10138265; doi:10.1371/journal.pone.0284157)
Supplement: S1 Appendix — (DOCX) [file pone.0284157.s003.docx]

**APPENDIX**

**Data of Studied Research**

| **State and federal territories** | **Serial**  **Number** | **Cumulative Covid-19 cases** | **Percentage of cumulative Covid-19 cases** | **Total population** | **Population density** | **Weighted population density** |
| --- | --- | --- | --- | --- | --- | --- |
| Selangor | 1 | 116078 | 33.60 | 6538100 | 822.299082 | 2308.114096 |
| Sabah | 2 | 54767 | 15.85 | 3908500 | 52.886177 | 351.653278 |
| Johor | 3 | 41255 | 11.94 | 3781100 | 197.281645 | 729.789194 |
| Sarawak | 4 | 16390 | 4.74 | 2816500 | 22.631579 | 169.697721 |
| Perak | 5 | 12966 | 3.75 | 2510300 | 119.674867 | 321.087502 |
| Kedah | 6 | 8435 | 2.44 | 2185200 | 230.214918 | 718.833158 |
| Kelantan | 7 | 6250 | 1.81 | 1906700 | 126.775266 | 744.940229 |
| Pulau Pinang | 8 | 16069 | 4.65 | 1773600 | 1690.753098 | 2457.822156 |
| Pahang | 9 | 4266 | 1.23 | 1678600 | 46.673154 | 89.842160 |
| Terengganu | 10 | 3593 | 1.04 | 1259200 | 96.601458 | 372.695235 |
| Negeri Sembilan | 11 | 17268 | 5.00 | 1128800 | 169.540403 | 411.664232 |
| Melaka | 12 | 6542 | 1.89 | 932700 | 544.801402 | 1093.941121 |
| Perlis | 13 | 330 | 0.10 | 254900 | 311.233211 | 558.724560 |
| WP Kuala Lumpur | 14 | 37820 | 10.95 | 1773700 | 7299.176955 | 8054.057018 |
| WP Labuan | 15 | 2328 | 0.67 | 99600 | 1082.608696 | - |
| WP Putrajaya | 16 | 1143 | 0.33 | 110000 | 2244.897959 |  |
